# Pyrrolizidine alkaloid-induced transcriptomic changes in rat lungs in a 28-day subacute feeding study

**DOI:** 10.1007/s00204-021-03108-x

**Published:** 2021-06-29

**Authors:** Julia Buchmueller, Heike Sprenger, Johanna Ebmeyer, Josef Daniel Rasinger, Otto Creutzenberg, Dirk Schaudien, Jan G. Hengstler, Georgia Guenther, Albert Braeuning, Stefanie Hessel-Pras

**Affiliations:** 1 German Federal Institute for Risk Assessment, Max-Dohrn-Str. 8-10, 10589 Berlin, Germany; 2 Institute of Marine Research (IMR), Postboks 1870 Nordnes, 5817 Bergen, Norway; 3 Fraunhofer Institute for Toxicology and Experimental Medicine ITEM, Nikolai-Fuchs-Straße 1, 30625 Hanover, Germany; 4 Leibniz Research Centre for Working Environment and Human Factors, Technical University Dortmund, Ardeystr. 67, 44139 Dortmund, Germany

**Keywords:** Pyrrolizidine alkaloids, Lung, Transcriptomics, Inflammation, Gene expression

## Abstract

Pyrrolizidine alkaloids (PAs) are secondary plant metabolites synthesized by a wide range of plants as protection against herbivores. These toxins are found worldwide and pose a threat to human health. PAs induce acute effects like hepatic sinusoidal obstruction syndrome and pulmonary arterial hypertension. Moreover, chronic exposure to low doses can induce cancer and liver cirrhosis in laboratory animals. The mechanisms causing hepatotoxicity have been investigated previously. However, toxic effects in the lung are less well understood, and especially data on the correlation effects with individual chemical structures of different PAs are lacking. The present study focuses on the identification of gene expression changes in vivo in rat lungs after exposure to six structurally different PAs (echimidine, heliotrine, lasiocarpine, senecionine, senkirkine, and platyphylline). Rats were treated by gavage with daily doses of 3.3 mg PA/kg bodyweight for 28 days and transcriptional changes in the lung and kidney were investigated by whole-genome microarray analysis. The results were compared with recently published data on gene regulation in the liver. Using bioinformatics data mining, we identified inflammatory responses as a predominant feature in rat lungs. By comparison, in liver, early molecular consequences to PAs were characterized by alterations in cell-cycle regulation and DNA damage response. Our results provide, for the first time, information about early molecular effects in lung tissue after subacute exposure to PAs, and demonstrates tissue-specificity of PA-induced molecular effects.

**Supplementary Information:**

The online version contains supplementary material available at 10.1007/s00204-021-03108-x.

## Introduction

**Fig. 1:**
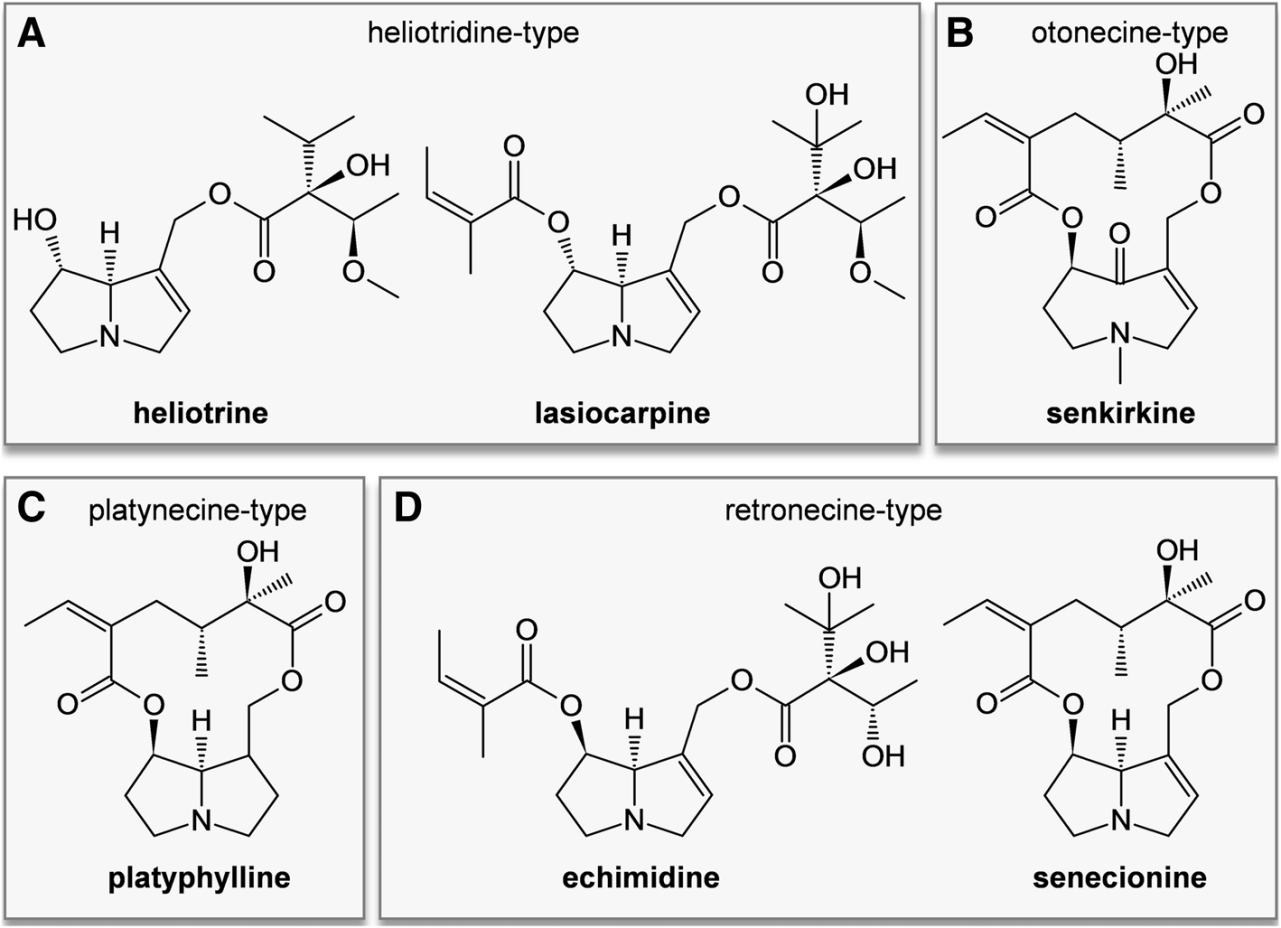
Structures of the PAs echimidine, heliotrine, lasiocarpine, senecionine, senkirkine and platyphylline. The PAs represent all structure types and different esterification

Pyrrolizidine alkaloids (PAs) are secondary plant metabolites occurring in approximately 3% of all flowering plants worldwide. The phytotoxins can be found for example in *Senecio jacobeae*, which is resident in Western Europe, South Africa, Australia, New Zealand, and North America (Stegelmeier et al. 1999). Furthermore, many other plants from families like Boraginaceae (all genera), Asteraceae (tribes Senecioneae and Eupatorieae) and Fabaceae contain PAs (Wiedenfeld et al. 2008), which are produced as a defense mechanism against herbivores. They are synthesized in an estimated number of 6000 plant species and show a huge structural variety (EFSA 2011; Teuscher and Lindequist 2009). PAs in general consist of a heterocyclic system of 1-hydroxymethyl pyrrolizidine esterified with one or two monocarboxylic or dicarboxylic acids. According to the structure of the necine base, PAs can be divided into four groups (retronecine-, heliotridine-, otonecine-, and platynecine-type) (Fig. 1) (Mattocks 1968). The platynecine group differs insofar that the PAs belonging to this group do not possess the 1,2-unsaturated double bond playing an important role in the bioactivation during the metabolism of PAs (Fig. 1C). Therefore, platynecine-type PAs are considered to be non-toxic. The PA-parent compounds are not bioactive themselves. They are metabolized and thus activated by cytochrome P450 monooxygenases (CYPs) (Stegelmeier et al. 1999; Mattocks 1968). Upon this bioactivation, the formed metabolite undergoes dehydration towards an instable pyrrolic ester, which can form adducts with nucleophilic centers of proteins or DNA. Approximately half of the PAs are assumed to be toxic, exhibiting cytotoxic, genotoxic and/or carcinogenic potential (Fu et al. 2004, 2014; Stegelmeier et al. 1999). Health of wild animals, livestock, and humans can be affected upon ingestion of contaminated feed or food, like herbs, tea, and honey (BfR 2020). The liver is most affected. Here, PA exposure predominantly leads to the hepatic sinusoidal obstruction syndrome (HSOS, formerly named veno-occlusive disease (Chen and Huo 2010; Kakar et al. 2010). HSOS is characterized by a prothrombotic hypofibrinolytic state due to damage of endothelial cells leading to a subsequent blockage of the central vein. Severe forms of HSOS can result in multiorgan failure (Gunther et al. 2019). PAs are also known to exert pneumotoxicity in rats and other species. Especially the PA monocrotaline is used as a model substance to induce lung injury in rats similar to human pulmonary arterial hypertension (PAH) (Hoorn and Roth 1992; Lame et al. 2005). Rats treated with monocrotaline show vascular remodeling along with increased pulmonary arterial pressure, which may result in right ventricular hypertrophy and death. Moreover, monocrotaline-induced PAH is characterized by an infiltration of lung tissue by macrophages and with signs of inflammation (Hoorn and Roth 1992; Zhuang et al. 2018). Due to the many possible sources of exposure, and the associated potential intoxication of humans and livestock, PAs represent an important research topic. Additionally, the currently existing risk assessment of PAs based on chronic, acute or subacute animal studies focuses predominantly on effects in the liver as the most affected organ (BfR 2020; EFSA 2011; Huang et al. 2017; Mei et al. 2007). Thus, our study aimed to elucidate effects on the lung in a subacute rat study orally treated for 28 days with six structurally different PAs that were found to occur most frequently in spices and culinary herbs (Kaltner et al. 2020). Gene expression regulation in the lung was analyzed to elucidate early-stage effects of PA intoxication. The PA representatives echimidine, heliotrine, lasiocarpine, senecionine, senkirkine, and platyphylline were used to monitor possible structure-specific PA effects.

## Materials and methods

### Chemicals

The structurally different PAs echimidine (94% purity), heliotrine (91% purity), lasiocarpine (98% purity), senecionine (99% purity), and senkirkine (98% purity) were purchased from PhytoLab (Verstenbergsgreuth, Germany). Platyphylline (95% purity) was purchased from BOC Sciences (New York, USA). PAs were dissolved in 0.15 M NaCl. Due to the poorer solubility, senecionine was first dissolved in 0.2 M hydrochloric acid, then the pH was adjusted to 6–7 and afterwards NaCl was added to a final concentration of 0.15 M.

### In vivo study

All animal experiments were conducted in compliance with the regulations of the German Animal Protection Law (Tierschutzgesetz of July 4, 2013) by following the Principles of Good Laboratory Practice (OECD, January 21, 1998 and German Chemicals Law, §19a, Appendix 1, July 2, 2008). The animal experiments were done at the Fraunhofer ITEM, Hanover, Germany. The PA treatment dosing scheme (0.1–0.33–1–3.3 mg/kg body weight/day) based on results of a National Toxicology program (NTP) study (NTP 2003).

Study design and animal treatment have already been described in Ebmeyer et al. 2020 and are summarized in more detail in the supplemental material.

### Gene expression analysis

#### RNA isolation and sample preparation

Frozen samples were pulverized with the help of mortar and pestle cooled with liquid nitrogen. The samples were transferred to RLT buffer (Qiagen, Hilden, Germany) with 1% β-mercaptoethanol. The RNA was extracted according to the manufacturer’s instructions via the RNeasy mini kit (Qiagen, Hilden, Germany) with the additional use of QiaShredder (Qiagen, Hilden, Germany). Quality and quantity of the RNA were checked with an Agilent 2100 Bioanalyzer with an RNA Nano Lab chip and a Tecan M200 Pro spectrophotometer with a NanoQuant Plate at 260 nm and 280 nm. For the quality measurements, it was verified that the samples had RNA integrity numbers (RIN) higher than nine as well as A260/A280 ratios of 1.8–2 and A260/A230 ratios higher than two. 300 ng RNA of each sample was shipped. The samples were labeled with a GeneChip WT Plus labeling kit at Eurofins (Aarhus, Denmark, Comp. No. 7230-GT0028). The microarray analysis on Affymetrix Clariom S rat array (Thermo Fisher Scientific, Waltham, Massachusetts, USA) was performed at Eurofins Genomics Europe Genotyping A/S (Eurofins, Aarhus, Denmark). The raw data of this publication have been deposited in NCBI’s Gene Expression Omnibus and are accessible through GEO Series accession number GSE166195.

#### Bioinformatical analysis

Data processing and statistical evaluation were performed in the R environment (R Core Team 2018). The raw data were normalized and summarized using the RMA (robust multi-array average) algorithm from the R package oligo version 1.54.1. The genes were associated with vendor-provided annotation information [Release 36 (Thermofisher)] and control genes were removed after background correction. Moreover, irrelevant genes with low or unchanged expression were removed (median < 4 or CV < 0.02, respectively) so that 12,570 and 12,066 genes remained for lung and kidney data, respectively. Differential gene expression analysis was performed by the R package limma version 3.46.0 (Ritchie et al. 2015). Linear models were fit and moderated *t*-statistics were computed by the eBayes function. Afterwards, the Benjamini–Hochberg correction was applied to exclude false-positive results, and thus differentially expressed genes with an adjusted *p *value < 0.1 were considered significant. This subset was subjected to principal component analysis (PCA) by the R package stats version 4.0.3. Heatmaps were generated by the R package ComplexHeatmap version 2.6.2 (Gu et al. 2016) using default settings if not mentioned otherwise. Intersections of gene sets were visualized as UpSet plots using R package UpSetR version 1.4.0.

For functional interpretation of differential gene expression results, lists with significant genes were subjected to Ingenuity Pathway Analysis (IPA, version 60467501, Qiagen Bioinformatics, Redwood City, California, USA). Comparison analysis to predict diseases and functions were performed and the resulting *z*-score was visualized as heatmaps. To compare gene expression changes across organs (lung, kidney and liver) one-way analysis of variance (ANOVA) was performed to extract the top 100 genes that were most affected in their expression by PA treatment per organ. Gene ontology (GO) Biological Process term enrichment analysis was performed using a hypergeometric test by the R package clusterProfiler version 3.16.1. (Yu et al. 2010), and false discovery rate (FDR) was applied to control for multiple testing. The data for liver samples were previously published by Ebmeyer et al. (2020) and data are accessible through GEO Series accession number GSE149678. The complete results of differential gene expression analysis in lung and kidney are summarized in the Supplementary data, Table S5.

The verification of gene expression analysis by quantitative reverse transcriptase polymerase chain reaction (RT-qPCR) is described in detail in the supplemental material.

## Results

Male Fisher rats were treated daily by oral gavage for 28 days with 3.3 mg of six structural different PAs/kg body weight. General observations, histological aberrations in livers, and liver transcriptomics data are reported elsewhere (Ebmeyer et al. 2020). The histological examinations revealed no changes in liver morphology. However, the gene expression data showed affected pathways dealing with DNA damage response and cell-cycle regulation. The present study elucidates structure-dependent effects of PAs on the development of lung toxicity by investigating PA-induced changes in lung-histology and pulmonary gene expression. Furthermore, the study reports transcriptional changes in kidneys and finally compares the organ-specific PA-induced transcriptional changes observed in liver, lung, and kidney.

### Body weight, organ weight and histopathological examination

As described in Ebmeyer et al. (2020), the total body weight of the animals did not change over the treatment period in comparison to the control animals. The relative weight of the lungs did also not show any significant changes (Supplementary data, Figure S1). For histopathological examination, lung slides were stained with hematoxylin and eosin as shown in Figure S2 in the supplemental material. The arrows highlight signs of perivascular mononuclear cell infiltration whereas black triangles indicate multifocal mixed inflammatory cell in the alveoli in the vicinity of the terminal bronchi. These lesions represent commonly found non-specific background effects often observed in rat lungs and were similarly observed in treated animals and controls. Taken together, body and organ weight observations, as well as histopathological findings indicate that PA at the doses applied did not induce histopathologically detectable changes at the organ level. In the following, more subtle molecular effects caused by PA-exposure are described.

### Transcriptomics analysis in the lung

**Fig. 2:**
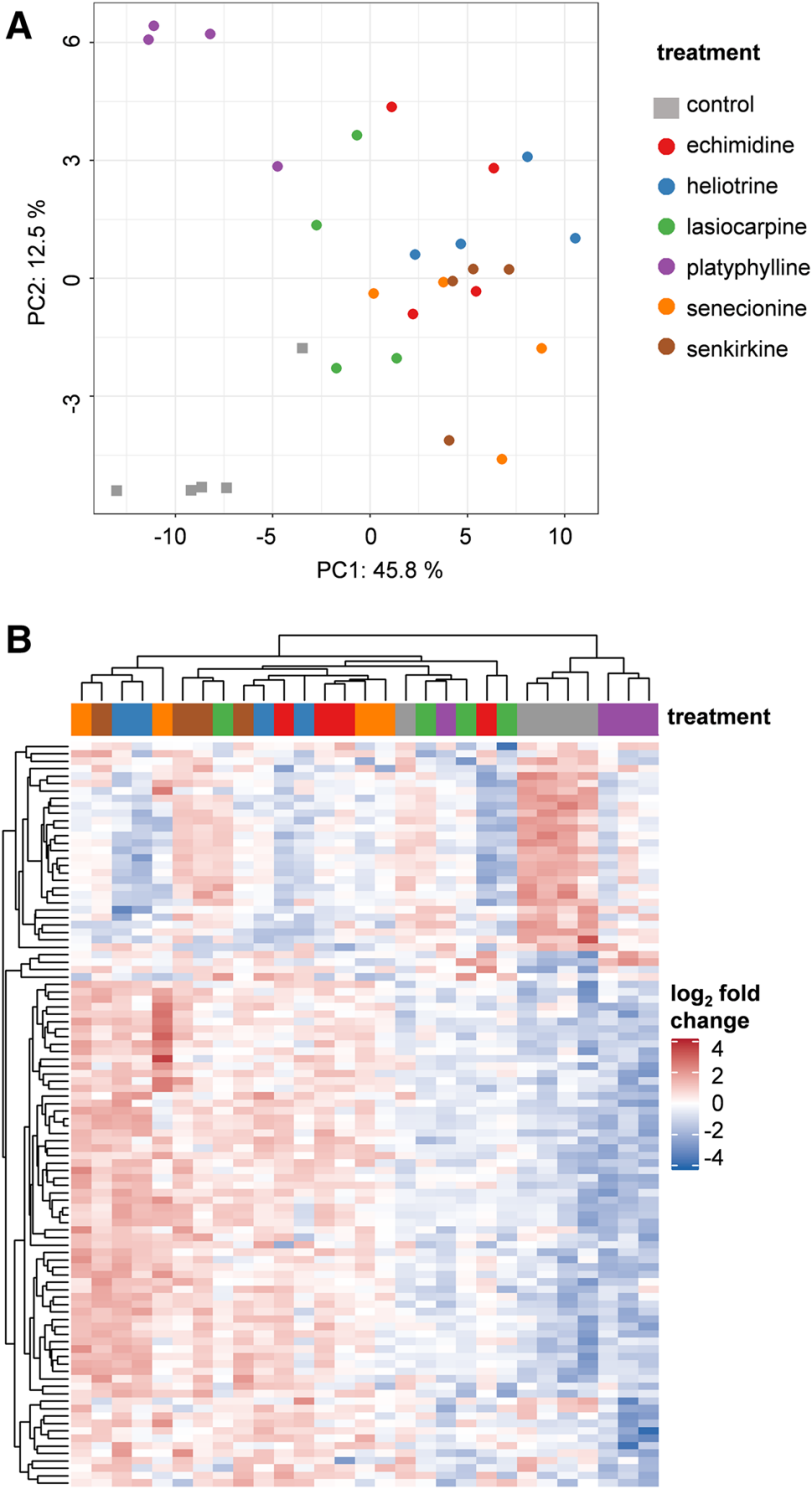
PCA scores plot and heat map of deregulated genes in rat lungs after treatment with six different PAs in comparison to the vehicle control. Rats were treated orally for 28 days with PAs at doses of 3.3 mg/kg body weight/day. The RNA of lungs was extracted and used for a whole genome microarray analysis with Affymetrix Clariom S rat arrays. The expression data were normalized and differential gene expression analysis was applied to identify 162 significantly deregulated genes. **A** The PCA scores plot depicts the expression variation of the different treatment groups as indicated by the colors. Each dot represents one animal. **B** The heat map shows the expression of the 162 differentially expressed genes with their scaled expression. Hierarchical clustering was performed according to the similarities in scaled gene expression (average linkage clustering method based on Pearson distance). Each column represents one animal. Upregulation of gene expression in comparison to the mean is depicted in red color whereas downregulation is depicted in blue color

**Fig. 3:**
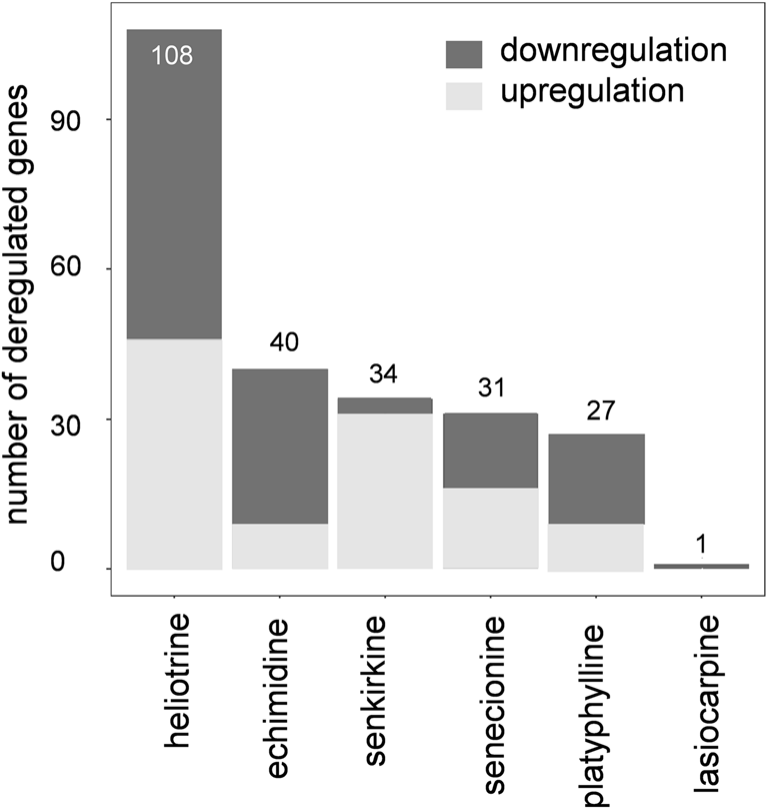
Bar plot of the number of deregulated genes in lungs from rats treated by gavage with six different PAs for 28 days, as compared to control conditions. The deregulated genes were determined by differential gene expression analysis (adjusted *p* value < 0.1) of microarray data from RNA of the treated animals. The data was normalized and corrected beforehand (see materials and methods section for details)

**Fig. 4:**
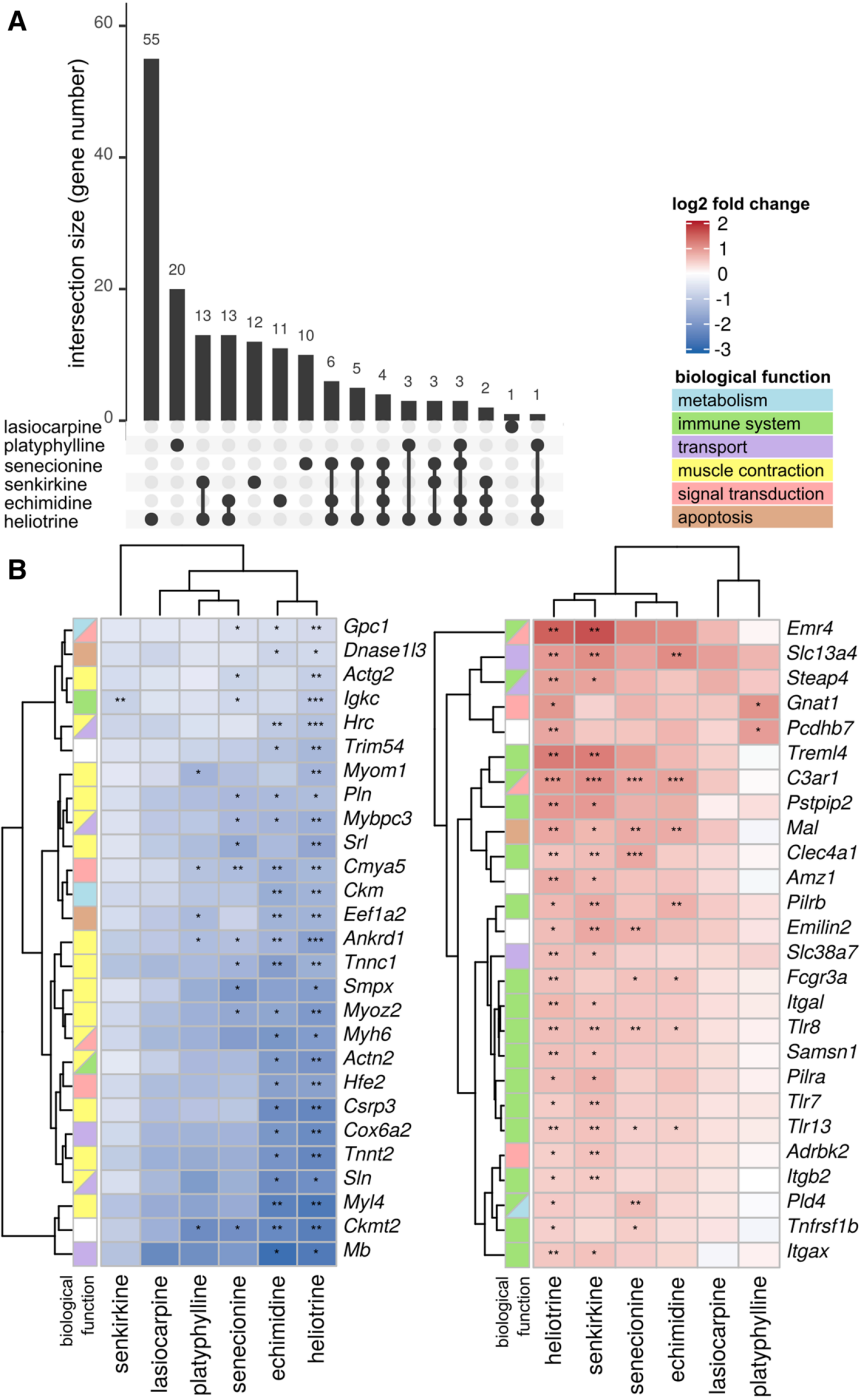
Commonly deregulated genes in lungs from rats exposed to different PAs. Rats were treated for 28 days by oral gavage with six structurally different PAs. Gene expression was analyzed using whole-genome microarray. After statistical evaluation, the significantly deregulated genes (*p*_adj_ < 0.1) were evaluated for commonly deregulated genes across the different treatment groups. In total, 53 genes revealed a significant deregulation in more than one treatment group. **A** The UpSet diagram visualizes the number of commonly deregulated genes in more than one treatment groups. Thereby, the numbers represent the number of deregulated genes in the specific intersection. The dots show in which treatment groups this common gene deregulation occurred. **B** The heatmap shows the expression of the 53 genes with significant deregulation in more than one treatment group. Upregulation is depicted in red; downregulation in blue. Significance of gene expression changes is indicated as follows: * *p*_adj_ < 0.1, **p_adj_ < 0.05, ***p_adj_ < 0.01

**Fig. 5:**
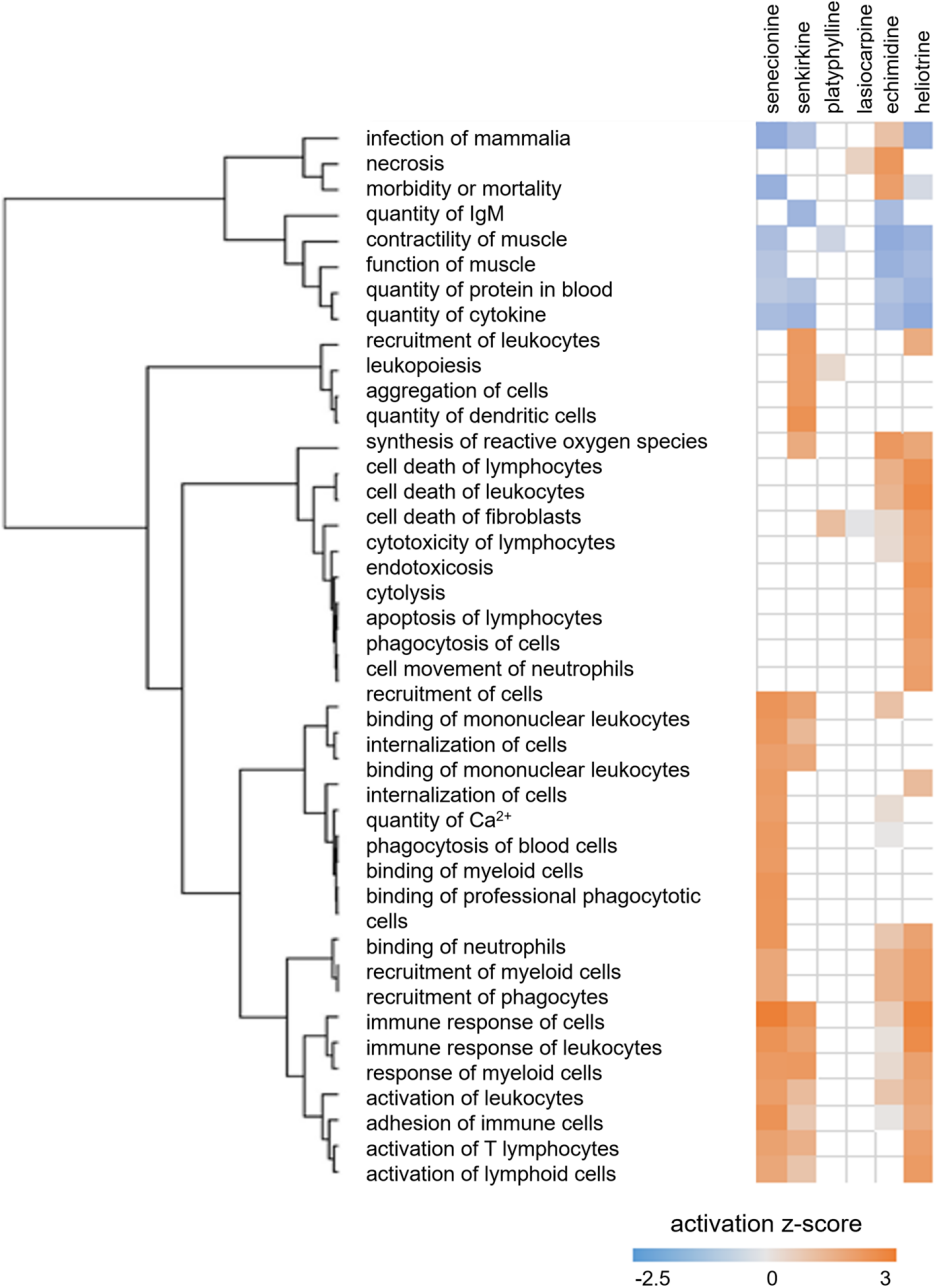
Predicted diseases and biofunctions in rat lungs due to treatment with six structurally different PAs. The significantly differentially expressed genes (*p*_adj_ < 0.1) were used for functional analysis with Ingenuity Pathway analysis (IPA) software. The software compares the data with literature data and predicts the probably influenced diseases and biofunctions. The prediction is based on the IPA activation *z*-score. Depicted are the activation *z*-scores (*z*-score cut off = 2) of the 30 most influenced diseases and biofunctions. The predicted pathways are hierarchically clustered. The complete list of predicted pathways can be found in Supplementary Table S3

Altered gene expression patterns in rat lungs after treatment with PAs were identified using whole-genome microarrays. The analysis was performed with RNA isolated from lungs of four animals treated with 3.3 mg echimidine, heliotrine, lasiocarpine, senecionine, senkirkine or platyphylline per kg body weight. The microarray data were processed as described in the material and methods section. A PCA plot and a heatmap of the subset of dysregulated genes in rat lungs after treatment with six different PAs are shown in Fig. 2. The PCA scores plot (Fig. 2A) and the heat map with hierarchical clustering (Fig. 2B) revealed that the control group and the platyphylline-treated group show clear differences in comparison to the other treatment groups analyzed in the present study. Whereas, the control and the platyphylline-treated groups separate among PC2, these two treatment groups differ from all other groups on PC1. Animals treated with lasiocarpine tend to cluster more with the control and platyphylline-treated animals than the other treatment groups. For the other treatment groups, namely echimidine, heliotrine, senecionine, and senkirkine, no clear clustering tendency was observed. The observations made based on the PCA scores plot were also visible in the heat map with hierarchical clustering. Differential gene expression analysis (*p*_adj_ < 0.1) revealed a total of 162 differentially expressed genes across all treatment groups (Fig. 3, the complete results are shown in Supplementary Table S5). The highest incidence of differentially expressed genes was detected in the lungs of animals treated with heliotrine, followed by the echimidine, the senkirkine and senecionine treatment groups. Animals treated with platyphylline or lasiocarpine showed only slight pulmonary gene regulation in all analyzed animals in this treatment groups. Subsequently, genes showing common regulation in expression in several treatment groups were examined in more detail (Fig. 4A). By comparison of gene regulation among the different treatment groups, a total set of 53 differentially expressed genes showed regulation in more than one treatment group (Fig. 4). The UpSet diagram shows the numbers of genes that are deregulated in one treatment group (indicated by individual dots below the graph) and the numbers of genes commonly deregulated in different treatment groups (connected dots). The numbers of genes only deregulated in one single treatment were highest for heliotrine (55 of 108) and platyphylline (20 of 27). This observation is reflected in the PCA scores plot (Fig. 2A), where platyphylline (PC1) and heliotrine (PC2) show the highest difference from the control and from each other. A total number of seven genes showed deregulation in four treatment groups (*C3ar1*, *Mal*, *Tlr8* and *Tlr13* by echimidine, heliotrine, senecionine, and senkirkine; *Ckmt2*, *Ankrd1*, *Cmya5* by echimidine, heliotrine, senecionine, and platyphylline). The cluster map furthermore illustrates the statistical significance of commonly deregulated genes. Interestingly, for the downregulated genes, the treatment of rats with echimidine and heliotrine induced the most pronounced deregulation. On the other hand, for the upregulated genes, heliotrine and senkirkine showed a higher number of significantly deregulated genes than the other treatment groups. The downregulated genes *Actn2*, *Ckm*, *Cmya5*, *Csrp3*, *Hrc, Mybpc3*, *Myh6*, *Myl4*, *Myom1*, *Myoz2*, *Pln*, *Sln*, *Smpx*, *Srl*, *Tnnc1*, *Tnnt2* and *Trim54* are, among others, involved in proper muscle function. Other downregulated genes were found to be involved in oncogenesis (*Ankrd1, Eef1a2*), immune response (*Ankrd1*) or apoptosis (*Dnase1*). Elucidating the functions of upregulated genes, the most prominent functions affected comprise cancer development and immune response including inflammatory processes. The expression of two genes encoding toll-like receptors (*Tlr7* and *Tlr8*) known to be involved in the innate immune system (Souyris et al. 2018) was upregulated. Furthermore, gene expression of the integrins *Itgal*, *Itgb2* and *Itgax* responsible for the regulation of development, immune response, cancer development, and homeostasis (Hynes 2002) was upregulated. Moreover, the genes *Fcgr3a*, *Pilra* and *Pilrb* encode for parts of the immunoglobulin G receptor or immunoglobulin-like receptors. Cancer development and progression was seen to be affected by *Tnfrsf1a, Adrbk2*, *Pld4*, *Gnat1* and *Samsn1* in other studies (Greco et al. 2015; Gao et al. 2017; Sang et al. 2016; Jiang et al. 2017; Kanda et al. 2016; Noll et al. 2014). The genes *Emr4*, *Steap4*, *C3ar1*, *Pstpip2* and *Clec4a1,* which were also upregulated due to PA treatment, were found to be involved in the regulation of inflammatory reactions (Stacey et al. 2002; Moldes et al. 2001; Scarl et al. 2017; Brennan et al. 2019; Yao et al. 2018). Furthermore, the mRNA levels of the transporters *Slc38a7* and *Slc13a4* were identified to be increased after PA treatment. In general, the Slc transporter family is responsible for the transport of essential substances and metabolites into cells and therefore plays a major role in cellular maintenance (Zhang et al. 2019). For verification of the whole genome microarray data set, five genes differentially expressed upon PA treatment were randomly chosen, and their gene expression was analyzed in the same RNA sample as for the microarray with RT-qPCR (Supplementary data, Figure S3). The tendencies of gene expression changes observed in the whole genome microarray could be reproduced by RT-qPCR. A more global view and interpretation of the detected gene expression patterns can be achieved by using Ingenuity Pathway analysis (IPA). Data of differentially expressed genes were loaded into IPA and used for predictions regarding the diseases and biofunctions affected by PAs in the lung (Fig. 5). IPA analysis of the 30 most influenced biofunctions and thus resulting diseases revealed that the PAs lasiocarpine and platyphylline did not lead to predicted alterations, whereas for the other PAs several influenced biofunctions were identified, with the immune response being predominantly affected. Moreover, pathways involved in proper muscle function were predicted to be altered. The complete IPA prediction can be found in Supplementary Table S3.

### Transcriptomics analysis in the kidney

Gene expression was also investigated in the kidney. Therefore, the kidneys from rats treated with 3.3 mg/kg body weight of the representative PAs senecionine or senkirkine showing high effects in the gene regulation in the liver were analyzed exemplarily as described in the materials and methods section. The data evaluation and statistical workflow were the same as for the lung samples. The evaluation of gene regulation in kidney samples revealed no significantly deregulated genes in comparison to the control group. Furthermore, a PCA showed no remarkable overall differences of gene expression in treatment groups in comparison to the control group (Supplementary Figure S6).

### Comparison with the liver

**Fig. 6:**
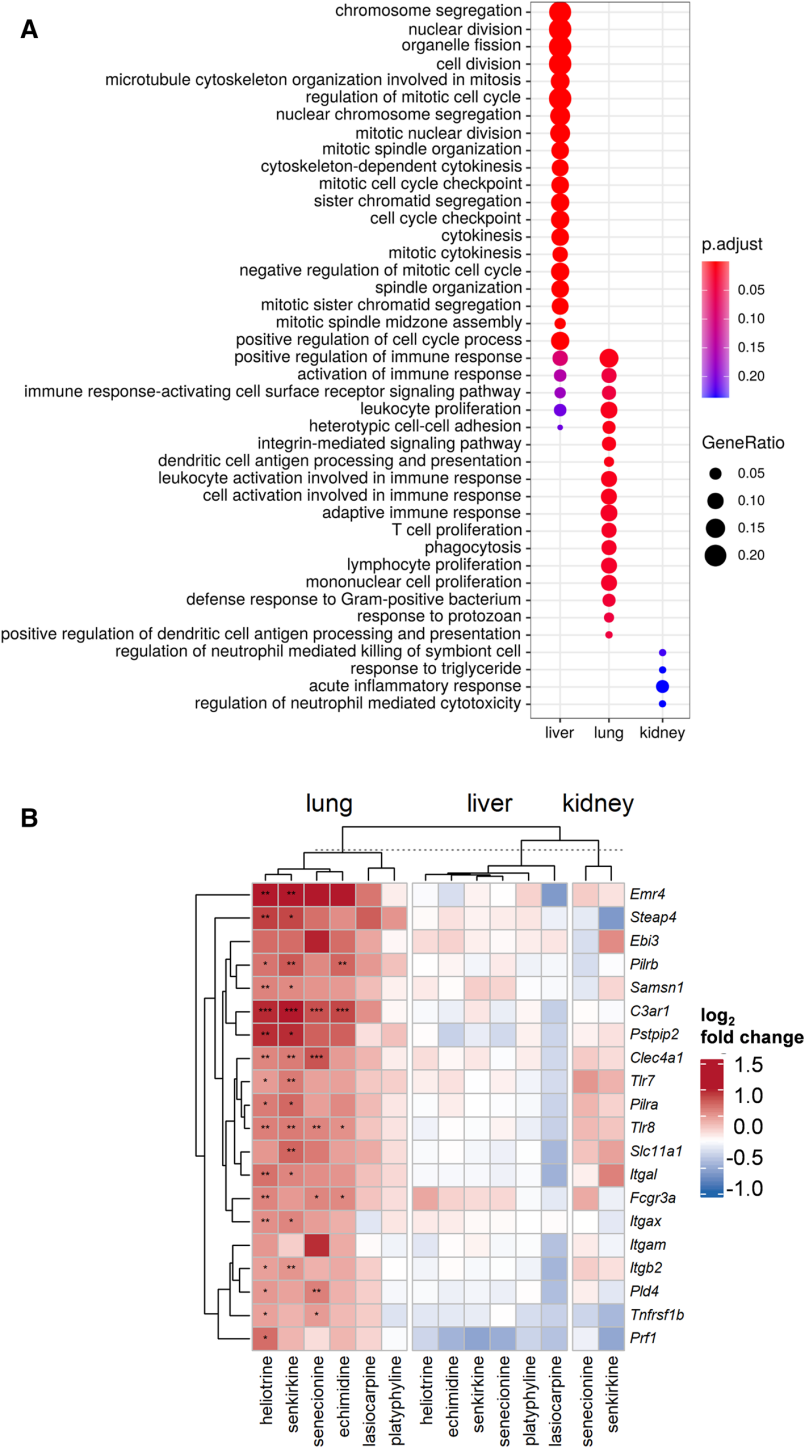
Comparison of gene deregulation in liver, lung and kidney in rats after treatment for 28 days with 6 different PAs. Rats were treated daily by oral gavage with 3.3 mg/kg body weight of the respective PA. RNA was extracted from the organs and whole-genome microarray analysis was performed. **A** GO term enrichment analysis was performed to compare the predictions of deregulated pathways in liver, lung, and kidney. The regulated pathways, dependent on the adjusted *p* value and gene ratio, are depicted. **B** The heatmap shows chosen genes, on the basis of IPA prediction and GO term enrichment analysis involved in immune response and inflammatory responses, with their logarithmic fold change in comparison to the control groups from liver, lung, and kidney. The deregulation of these genes is compared in the three organs according to their log2 fold change. The genes and PAs are hierarchically clustered

To gain better insight into the differences or similarities of the early molecular effects in the liver, lung, and kidney, we compared our data to a previously published liver data set from the same animals (Ebmeyer et al. 2020). ANOVA was performed for each data set to extract the top 100 genes that were most affected by PA treatment per organ. Subsequently, GO term enrichment analysis was applied based on these gene sets to compare PA effects in liver, lung, and kidney. The GO term enrichment plot depicts that PA-induced deregulation of gene expression results in different patterns of deregulated pathways in the liver, lung, and kidney (Fig. 6A). The microarray analysis and data evaluation of the liver revealed that mostly pathways involved in cell-cycle regulation and DNA damage response were regulated upon PA treatment (Ebmeyer et al. 2020). In contrast, gene expression analysis in the lung showed more regulation in pathways associated with an immune and inflammatory response. For the kidney samples, gene expression data was used to detect tendencies of deregulation even if the regulation was not statistically significant. Here, the most prominent pathways affected after PA treatment were inflammatory response and immune response. Overall, the enrichment plot allows the conclusion that PAs regulate remarkably different patterns of cellular pathways in the observed organs, with only minor overlaps for immune response between lung and liver. The heatmap in Fig. 6B shows the regulation of genes involved in immune response in the liver, lung, and kidney. The genes were chosen on the basis of the IPA prediction and GO term annotation. The depicted tendencies reveal that genes that are differentially regulated in the lung show no significant regulation in the liver and kidney. The same effect is observable when the expression of genes found to be regulated in the lung in more than one treatment group was compared with the expression of the same genes in the liver or vice versa (Supplementary Figures S4 and S5).

## Discussion

PAs can pose a threat to human health due to their toxic and/or carcinogenic potential. These secondary plant metabolites can occur in food, tea or in food supplements. Therefore, the uptake can accumulate over different food groups (BfR 2020). According to the literature, there have been cases of intoxication of two children with highly contaminated tea reaching a dose of 0.8–1.7 mg/kg body weight and 3 mg/kg body weight (Stillman et al. 1977; Fox et al. 1978). Concluding, the dose of 3.3 mg/kg body weight applied in this study represents a dose that can be reached in extreme cases. As described in the introduction, PAs can induce severe liver and lung toxicity and toxicological research is predominantly focused on PA-induced hepatoxicity. Renal excretion of most PAs (Swick et al. 1982) also makes the kidneys a putative additional target organ. Nevertheless, data of PA-induced effects in the kidney are lacking. For this reason, we additionally examined the gene regulation in this organ to detect possible early effects of toxicity. Several studies were performed to evaluate the effect of PAs on gene expression patterns in the liver (Mei et al. 2007; Ebmeyer et al. 2020); the latter, presenting results of a whole-genome microarray analysis of the livers of the same animals also examined in this study. A main outcome was that predicted regulated pathways were predominantly related to DNA damage response and cell-cycle regulation. However, only a few studies evaluated the effects of PAs in gene expression in the lung or in human lung cells. Most of the studies focused on monocrotaline since this PA is often used to induce PAH in rats (Xiao et al. 2020; Kishimoto et al. 2015; Fu et al. 2014). Therefore, the present study aimed to provide information which pathways are affected in rat lung after repeated-dose exposure to six structurally different PAs. After oral treatment of rats with six different PAs, each administered at a dose of 3.3 mg PA/kg body weight, we observed a change in transcription patterns in the lung especially for the PAs heliotrine and senkirkine (Fig. 3) that are completely different from the effects observed in the liver. IPA analysis of differentially regulated genes in the lungs predicted regulation of several pathways dealing with immune response and inflammation. Transcriptome analysis of monocrotaline-treated rats showed a strong linkage of the initiation and progression of PAH to inflammation markers after administration of a single dose of 40 mg monocrotaline/kg body weight (Xiao et al. 2020), as well as mitochondrial damage, angiogenesis, and fibrosis after administration of 60 mg monocrotaline/kg body weight (Potus et al. 2018). Furthermore, other studies showed that the response to injection of 60 mg monocrotaline/kg body weight was associated with p53 and HIF signaling pathways (Wang et al. 2019). Additionally, the progression and severity of PAH in humans is highly dependent on genetic predisposition. The gene *BMPR2* is named in several studies as an important factor in the progression of PAH in humans and also in the monocrotaline rat model of PAH (Garcia-Rivas et al. 2017; Tuder et al. 2013; Lane et al. 2000; Machado et al. 2015; Cheng et al. 2017). Nevertheless, also other genes were found to play a fundamental role in the initiation or progression of PAH in humans and rats: *BMP9*, *CAV1*, KV1.5, *KCNK3*, *EIF2AK4*, *ENG*, *SMAD9*, *ALK1* (as reviewed in: Wang et al. 2016; Austin et al. 2012; Marsboom et al. 2017; Yuan et al. 1998; Remillard et al. 2007; Navas 2017; Eyries et al. 2014; Tu et al. 2019; Ramos et al. 2008; Jasmin et al. 2006; Lee et al. 2018). However, the expression of these genes was not significantly deregulated in our study (Supplementary Table S2). The disturbance of BMP signaling and/or TGF-beta signaling pathways is a prominent feature of PAH (Olschewski et al. 2018; Hurst et al. 2017; Machado et al. 2015). Thus, we compared our gene expression data with these pathways using IPA. The analysis showed that only some genes involved in the TGF-beta signaling pathway were slightly but not significantly regulated in this study but no genes of the BMP signaling pathway were identified to be regulated in our data set. Hence, the PA-treated animals did not show any signs of PAH neither in histopathological examination nor in gene expression analysis. This phenomenon could be explained by the relatively low doses used in our study. In our study, the most substantial result is the predicted regulation of several pathways dealing with inflammation and immune response (Fig. 5). For additional verification of this finding, we investigated the deregulation of genes known to be involved in inflammatory processes in lungs from mice from another study treated with senecionine in a high dose, acute study (Supplementary Table S1). The investigated gene expression also revealed deregulation of the regarded inflammation marker in comparison to the control group. This shows that the gene expression analysis allows the detection of possible early events of PAH in our subacute and in our acute study (Tuder et al. 2013; Mamazhakypov et al. 2021). Additionally, the applied doses of PAs in our study induced gene expression changes of some genes associated with smooth muscle cells. This could be a first hint for PAH since disturbance of smooth muscle cells is a key factor in the pathobiology of pulmonary hypertension (Gao et al. 2017). In contrast to the other PAs, lasiocarpine showed the lowest number of significantly deregulated genes in the lung (Fig. 3) even though lasiocarpine is known to be one of the most potent PAs in the liver (Lester et al. 2019; Merz and Schrenk 2016). This may be associated with the fast hepatic metabolism of lasiocarpine resulting in predominant severe liver damage even though in the examined animals for this study this effect was not observable. Moreover, Geburek et al. (2020) showed that the metabolism rate varies strongly for different PAs by incubation with rat or human liver microsomes. PAs like heliotrine and senkirkine, showing low metabolism rates with liver microsomes led to more pronounced gene expression regulation in the lungs in the present study. It may thus be hypothesized that the metabolism rate in the liver is reverse proportional to the damage in the lung. The reactive metabolites can be transported via blood from liver to lung as shown by He and colleagues. The group found metabolites in plasma and red blood cells after oral administration of 120 mg/kg BW monocrotaline in mice (He et al. 2021). Gene expression analysis of the kidneys revealed no significant changes. This can be explained by the fact, that most PAs are excreted renally as more stable and soluble N-oxides which exert no toxic properties (BfR 2020). We demonstrate in the present study that repeated-dose application of 3.3 mg PA/kg body weight leads to molecular changes related to pulmonary pathobiology and lung inflammation, underlining the sensitivity of omics approaches to observe early subtle changes with high sensitivity, in the absence of manifest histopathologically observable effects.

## Supplementary Information

Below is the link to the electronic supplementary material.

Supplementary file1 (PDF 4270 kb)

Supplementary file2 (XLSX 5584 kb)

Supplementary file3 (XLSX 27 kb)

## Data Availability

The raw data of this publication have been deposited in NCBI’s Gene Expression Omnibus and are accessible through GEO Series accession number GSE166195.

## Abbreviations

ANOVA: One-way analysis of variance
CYP: Cytochrome P450 monooxygenase
FDR: False discovery rate
GO: Gene ontology
HSOS: Hepatic sinusoidal obstruction syndrome
IPA: Ingenuity pathway analysis
NTP: National Toxicology Program
PA: Pyrrolizidine alkaloid
PAH: Pulmonary arterial hypertension
RT-qPCR: Quantitative reverse transcriptase polymerase chain reaction
RIN: RNA integrity number
