# Supplementary material for: Pyrrolizidine alkaloid-induced transcriptomic changes in rat lungs in a 28-day subacute feeding study
Source: Arch Toxicol. 2021 Jun 29;95(8):2785–96. doi: 10.1007/s00204-021-03108-x (PMC8298252; doi:10.1007/s00204-021-03108-x)
Supplement: Supplementary file 1 — Supplementary file1 (PDF 4270 kb) [file 204_2021_3108_MOESM1_ESM.pdf]

-Supplementary material-

Pyrrolizidine alkaloid-induced transcriptomic changes in rat lungs in a 28-day subacute feeding study

Julia Buchmueller[a], Heike Sprenger[a], Johanna Ebmeyer[a], Josef Daniel Rasinger[b], Otto Creutzenberg[c], Dirk Schaudien[c], Jan G. Hengstler[d], Georgia Guenther[d], Albert Braeuning[a], Stefanie Hessel-Pras[a]

[a] German Federal Institute for Risk Assessment, Max-Dohrn-Str. 8-10, 10589 Berlin, Germany

[b] Institute of Marine Research (IMR), Postboks 1870 Nordnes, NO-5817 Bergen, Norway

[c] Fraunhofer Institute for Toxicology and Experimental Medicine ITEM, Nikolai-Fuchs-Straße 1, 30625 Hanover, Germany

[d] Leibniz Research Centre for Working Environment and Human Factors, Technical University Dortmund, Ardeystr. 67, 44139 Dortmund, Germany

Corresponding author:

Dr. Stefanie Hessel-Pras

ORC-ID: 0000-0002-6153-0035

German Federal Institute for Risk Assessment

Max-Dohrn-Str. 8-10

10589 Berlin, Germany

E-mail: stefanie.hessel-pras@bfr.bund.de

Tel.: +49-30-18412-25203

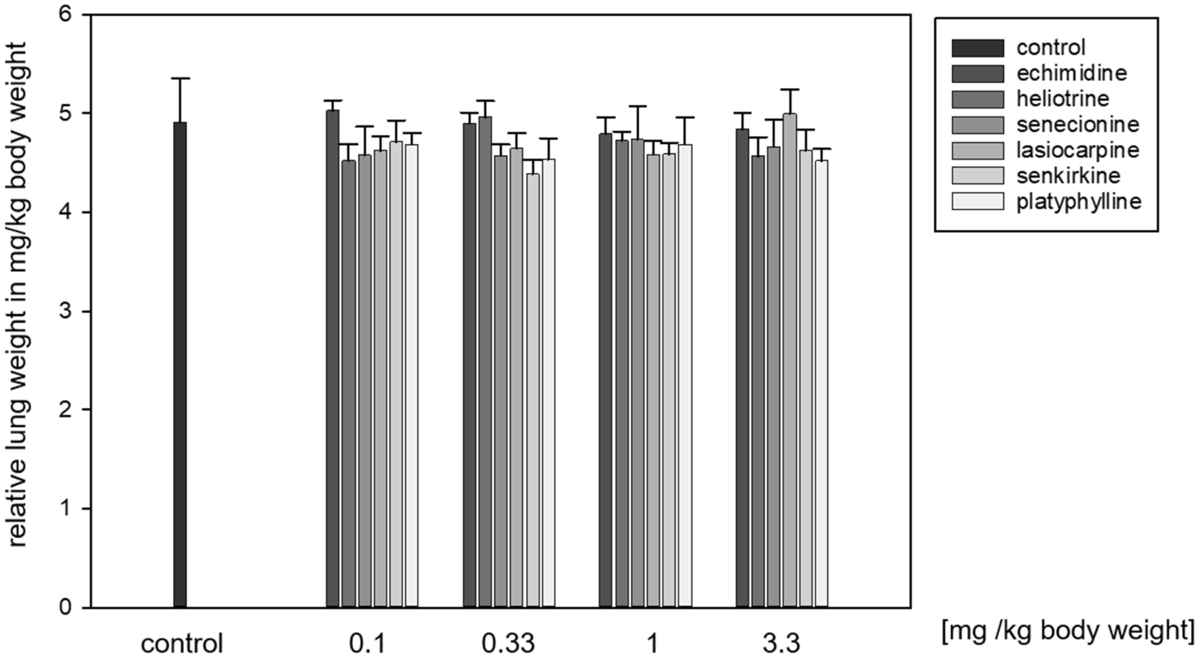

Figure S1: Relative lung weights of rats treated for 28 days with six different PAs. Rats were treated by gavage daily with 3.3 mg/ kg body weight of echimidine, heliotrine, senecionine, lasiocarpine, senkirkine or platyphylline for 28 days. After sacrifice, lungs were extracted and weighed. The bars show the relative organ weights in comparison to the control group.

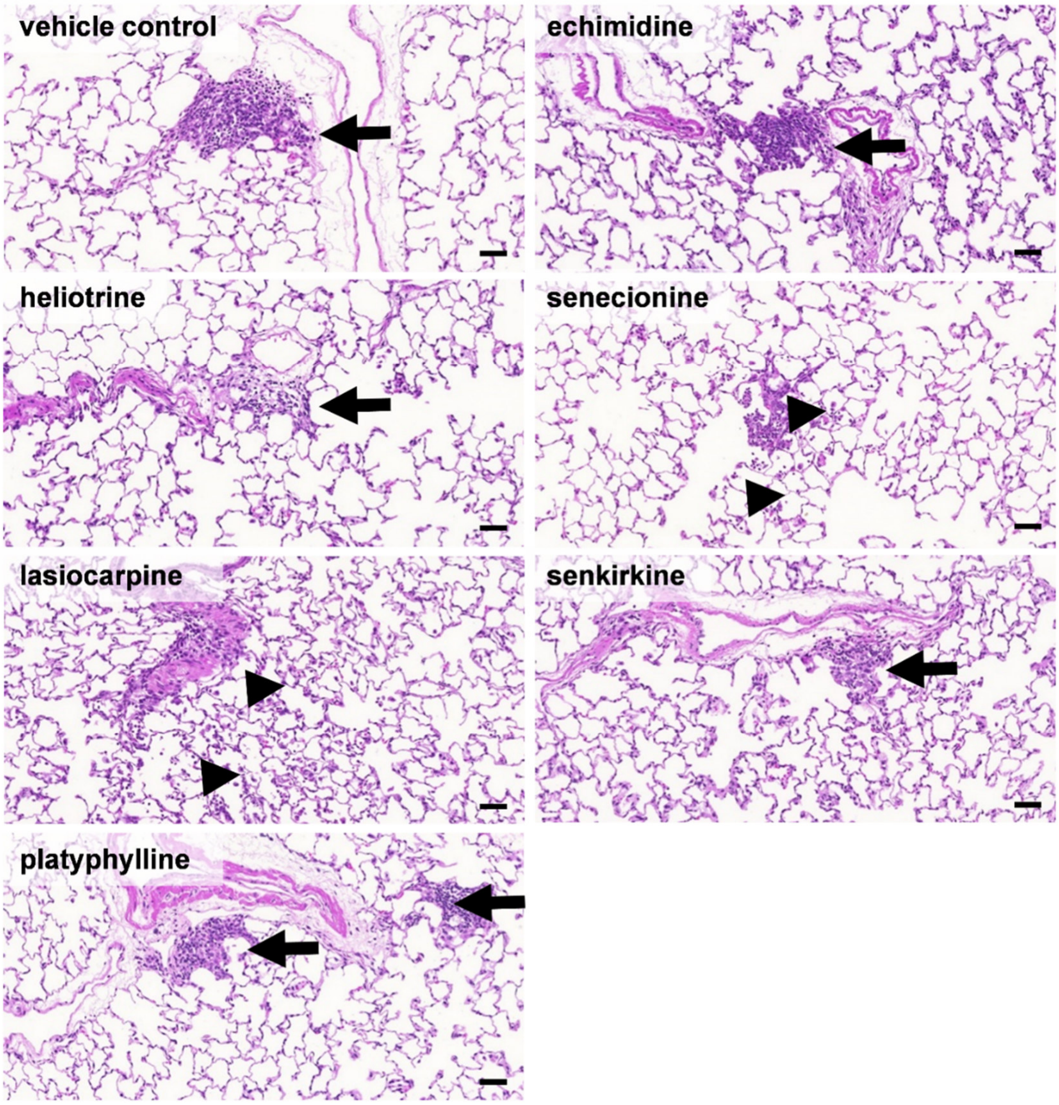

Figure S2: Histological examination of rat lung tissue sections of rats treated daily with 3.3 mg/kg body weight of six different pyrrolizidine alkaloids or vehicle control by oral gavage for 28 days. The lung sections were stained with hematoxylin and eosin. The arrows highlight signs of perivascular mononuclear cell infiltration. The black triangles indicate multifocal mixed inflammatory cells. Bar = 50 µm.

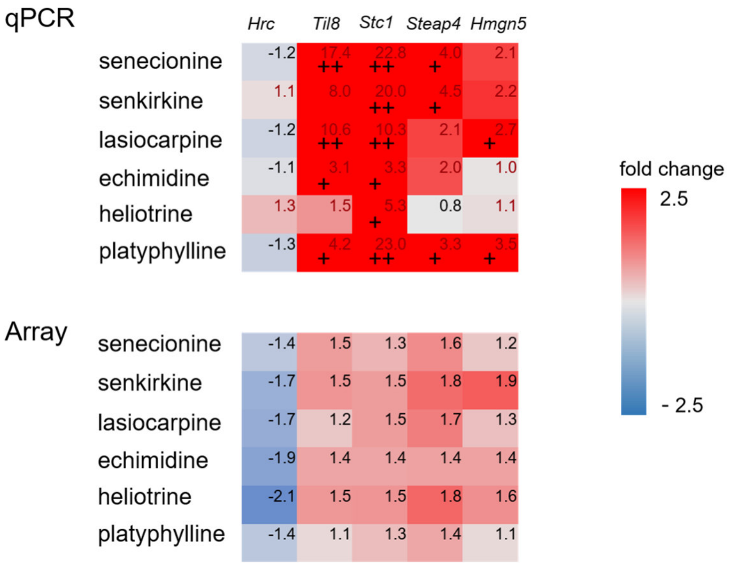

Figure S3: Verification of PA-induced regulation of gene expression. RNA from lungs of rats treated with 3.3 mg PA/kg body weight by gavage daily for 28 days was extracted and the expression of genes was investigated by microarray or qPCR. The qPCR was done by reverse transcribing the RNA into cDNA before adding the appropriate primers and SYBR Green/ROX mix for the qPCR. The results were normalized to housekeeping genes. Fold changes of relative gene expression from four animals are depicted. Fold change values that are out of the color range are additionally marked with + (+ = values up until 10, ++ = values up until 23).

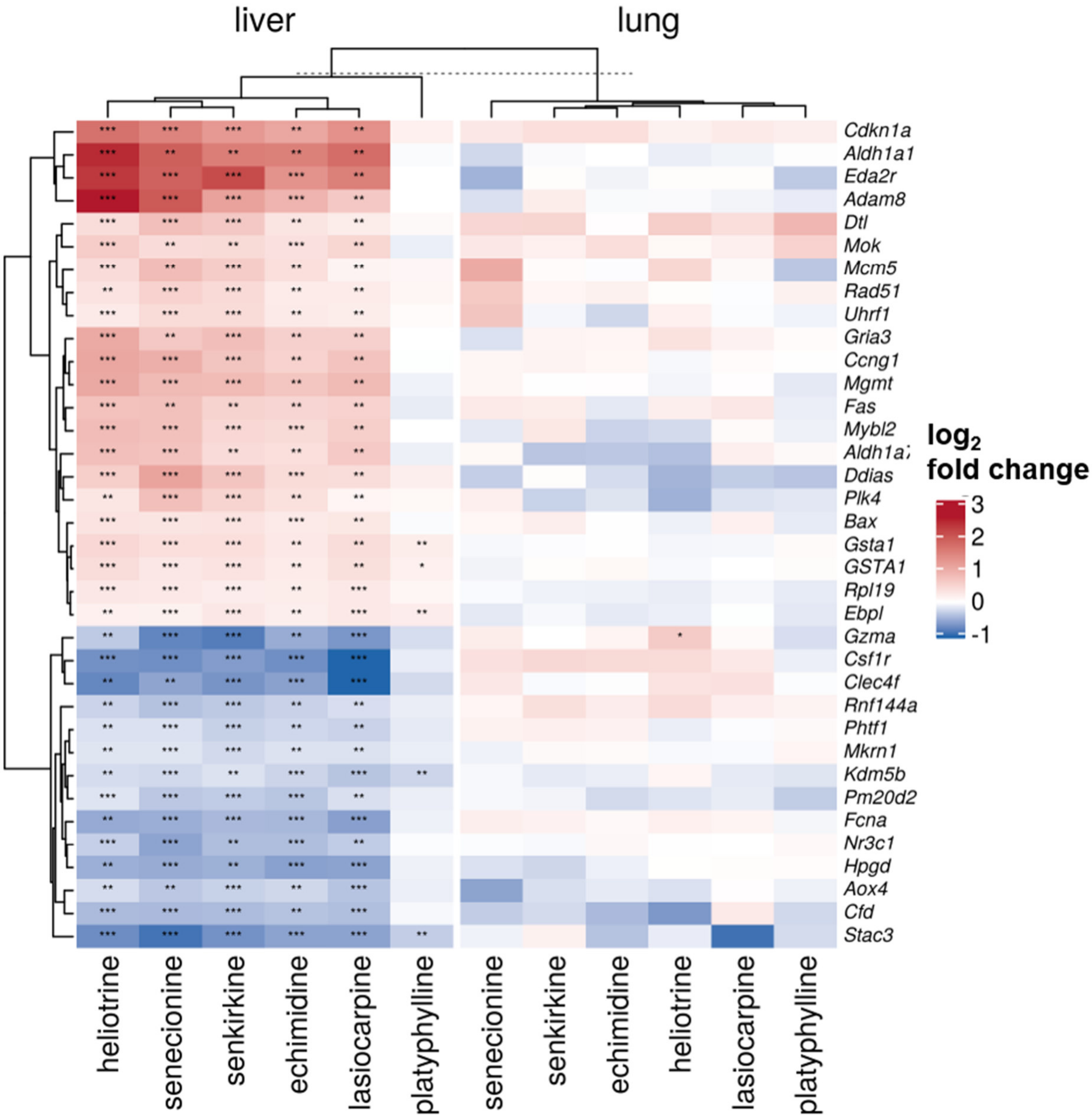

Figure S4: Comparison of gene regulation in liver and lung. Rats were treated by gavage for 28 days daily with 3.3 mg PA/kg body weight. The RNA of lungs was extracted and whole genome microarray analysis was performed. The data were compared to data previously published by Ebmeyer et al. (2020). They found 36 genes commonly significant regulated in all treatment groups in the liver (except for platyphylline). These genes were used for the comparison between the two organs. The heatmap shows the $\log_2$ fold change of this gene expression in both organs. Significance of gene expression alteration in the lung is indicated (*) with the definitions of *p<0.05, ** p<0.01, ***p<0.001.

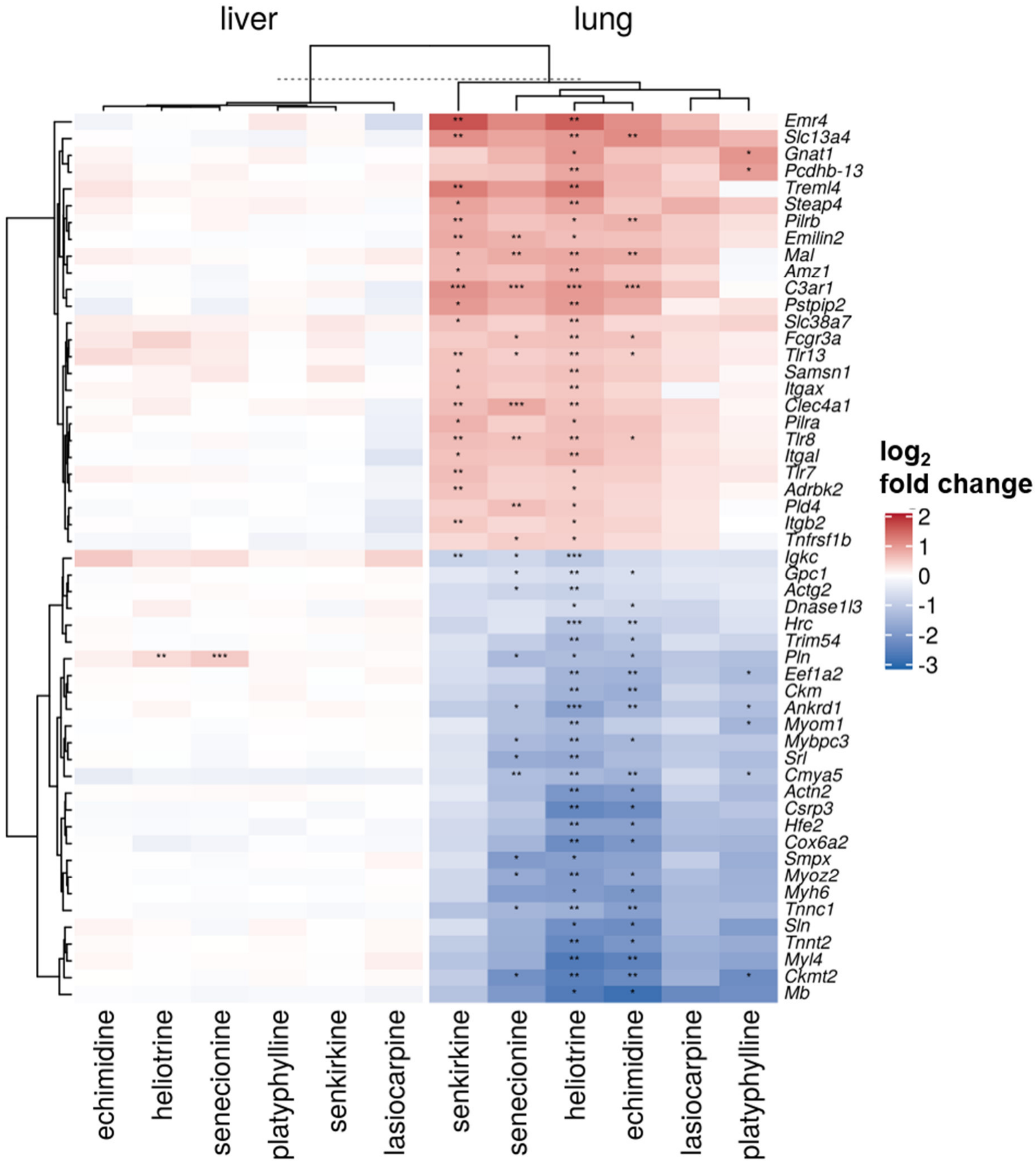

Figure S5: Comparison of gene regulation in lung and liver. Rats were treated by gavage for 28 days daily with 3.3 mg PA/kg body weight. The RNA of lungs was extracted before a whole genome microarray analysis was performed. The data were compared to data previously published by Ebmeyer et al. (2020). Thereby, genes showing significant regulation in two treatment groups in the lung were chosen. The heatmap shows the $\log_2$ fold change of the gene expression in the lung and liver. Significance of gene expression changes is indicated as follows: $p_{adj} < 0.1$, ** $p_{adj} < 0.05$, *** $p_{adj} < 0.01$.

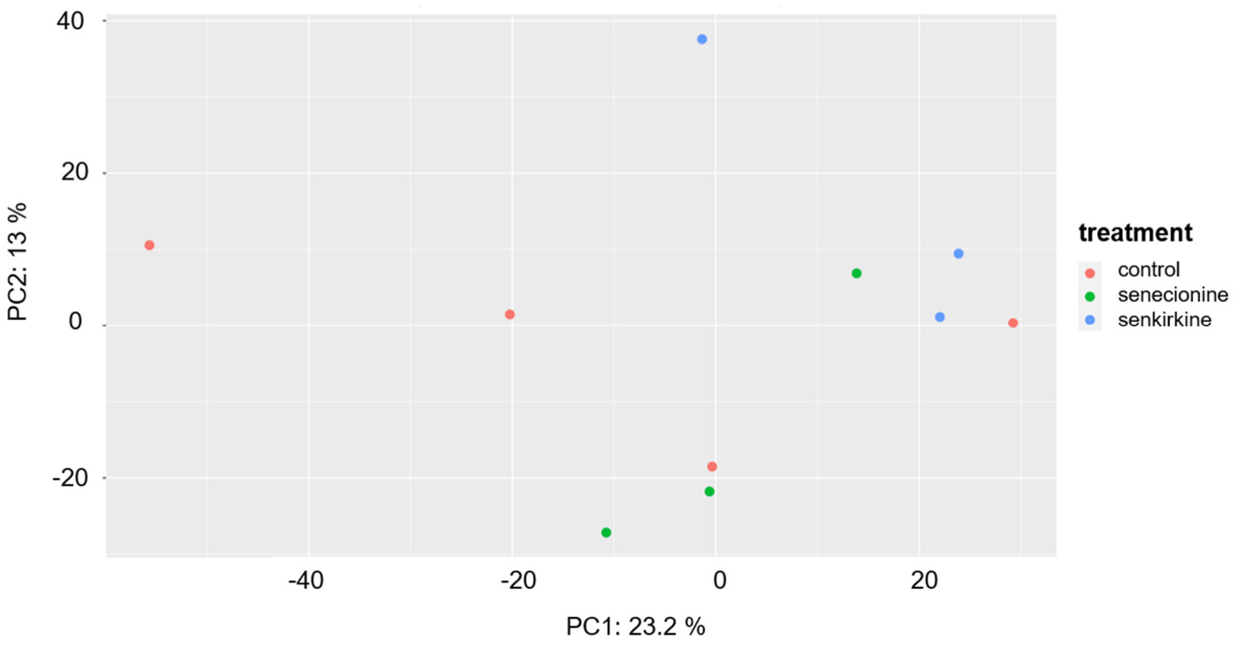

Figure S6: PCA scores plot of genes in kidneys from rats treated orally with 3.3 mg/kg body weight of senecionine or senkirkine for 28 days. The RNA was extracted from the kidneys before performing a whole genome microarrays analysis as described in material and methods section.

Table S1: Gene expression data of genes involved in inflammatory responses in mouse lungs. Mice were treated with different doses of senecionine for 36 h or with 60 mg/kg body weight for different time intervals as described in more detail in Hessel-Pras et al., 2020.

		[mg/kg body weight]		
	control	15	30	60
Cxcl12	1.0	1.8	-1.1	-1.4
Cxcl13	1.0	-1.2	-1.2	2.7
Lcn2	1.0	-4.0	-1.0	-2.9
Saa3	1.0	1.8	-1.2	-2.4

	[h]				
control	12	24	36	48	72
1.0	-1.2	-1.4	-1.9	-2.3	-2.0
1.1	2.0	-2.8	-1.2	-3.0	-1.2
1.1	-1.1	-2.4	-4.7	-2.4	-1.6
1.1	-1.6	-1.4	-2.4	-1.2	-1.2

fold change

2

-4

Table S2: Relative expression data for genes that are known to play a role in the progression of PAH. Rats were treated daily with 3.3 mg PA/kg body weight for 28 days. The RNA of the lungs was extracted and the gene expression was analyzed with microarray analysis as described in material and methods section.

gene	senecionine	senkirkine	platyphylline	lasiocarpine	echimidine	heliotrine
Kcnk3	0.6	1.0	1.0	0.9	0.9	1.0
Notch1	0.9	0.9	1.1	1.0	1.0	1.1
Smad9	0.9	1.1	1.0	1.1	1.0	1.1
Nos3	1.1	1.2	1.0	1.3	1.1	1.1
Sirt3	0.8	1.0	1.0	0.9	1.0	0.8
Agtr1a	0.9	0.9	1.0	1.0	1.1	1.1
Serpine1	1.6	1.2	1.1	1.1	1.1	1.1
Edn1	1.6	1.2	1.1	1.3	1.1	1.2
Tbx4	1.0	1.1	1.0	1.2	1.2	1.2
Trpc6	1.1	1.1	1.2	1.1	1.1	1.1
Thbs1	1.1	1.1	1.1	1.1	1.0	1.0

fold change

0.5

1.6

Table S3: Complete list of predicted diseases and biofunctions from IPA analysis. Rats were treated with 3.3 mg PAs per kg body weight for 28 days. RNA of lungs was extracted and used for micro array analysis. The significantly regulated genes were uploaded in IPA for the prediction of affected diseases and biofunctions. Indicated are the specific activation z-scores for each treatment.

diseases and biofunctions	senecionine	senkirkine	platyphylline	lasiocarpine	echimidine	heliotrine
Activation of leukocytes	2.0	1.8				1.9
Immune response of leukocytes	1.7	2.2			-0.3	2.4
Immune response of cells	1.9	2.4				2.6
Contractility of ventricular myocardium					-2.0	
Cardiac contractility	0.3				-1.7	-1.2
Blood pressure					-2.0	-1.4
Contractility of muscle	-0.8				-2.2	-2.0
Function of muscle					-2.0	-1.8
Contractility of cardiac muscle					-2.0	-1.7
Quantity of neutrophils		-1.6				-1.0
Quantity of phagocytes		-0.9				-1.7
Quantity of cytokine		-2.1				-2.4
Quantity of myeloid cells		-1.6				-1.6
Infection of mammalia		-1.7				-2.0
Quantity of protein in blood		-1.6				-2.0
Cell movement of leukocytes		1.1	-0.4			-0.4
Activation of cells	0.9	2.2				1.5
Inflammatory response	0.9	2.4				0.9
Response of myeloid cells		2.0			-0.2	2.0
Adhesion of immune cells		2.0				1.7
Activation of lymphocytes		1.3				2.0
Phagocytosis of cells		1.7				2.1
Activation of lymphoid cells		1.5				2.2
Activation of T lymphocytes		1.5				2.0
Aggregation of cells		2.2				
Cell movement		2.1				
Cell movement of neutrophils		2.1				
Recruitment of cells		2.0				
Aggregation of blood cells		2.0				
Cell movement of macrophages		2.0				
Activation of phagocytes		1.9				0.7

z-score
3
-2.5

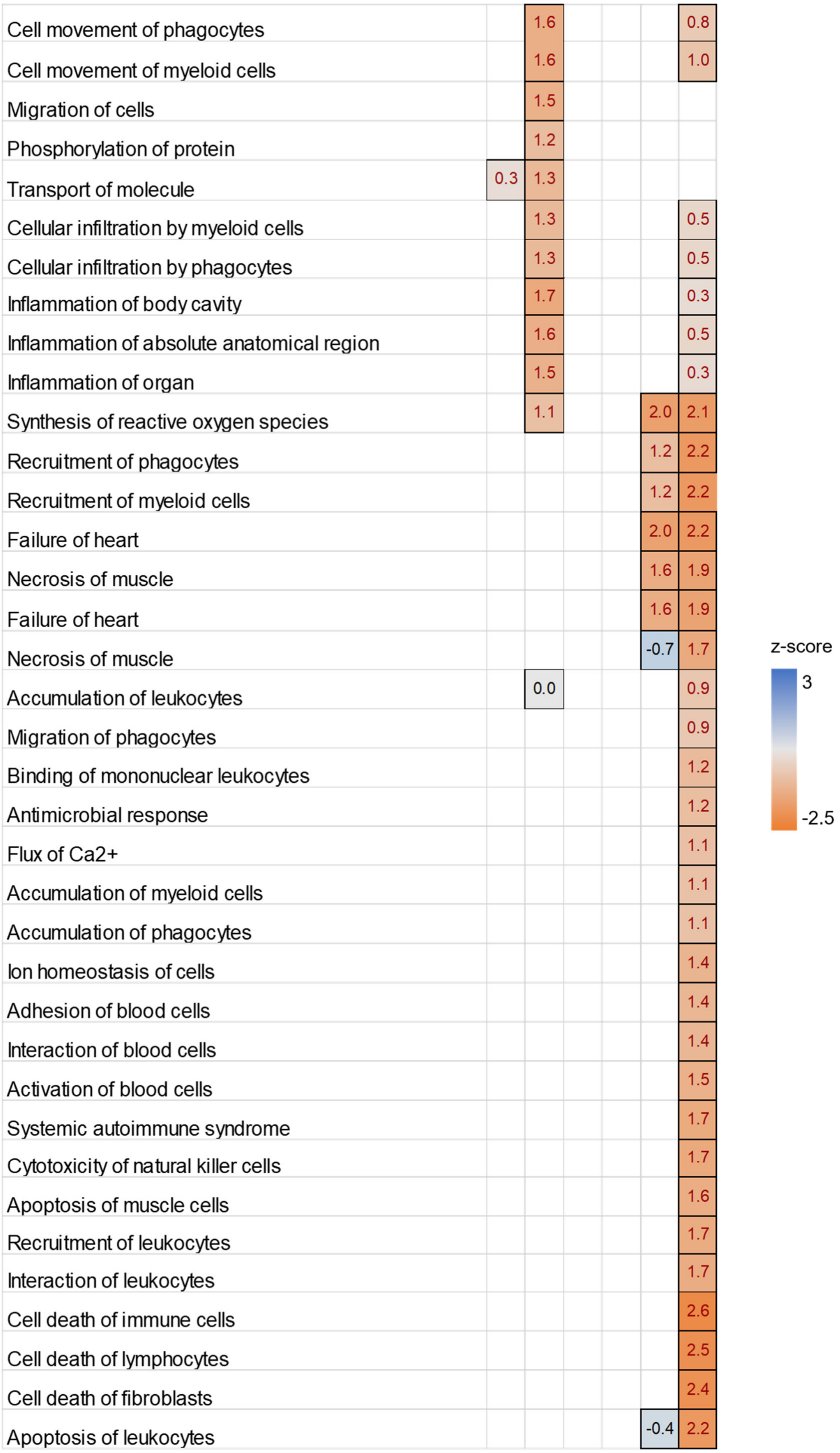

Cell movement of phagocytes 1.6 0.8
Cell movement of myeloid cells 1.6 1.0
Migration of cells 1.5
Phosphorylation of protein 1.2
Transport of molecule 0.3 1.3
Cellular infiltration by myeloid cells 1.3 0.5
Cellular infiltration by phagocytes 1.3 0.5
Inflammation of body cavity 1.7 0.3
Inflammation of absolute anatomical region 1.6 0.5
Inflammation of organ 1.5 0.3
Synthesis of reactive oxygen species 1.1 2.0 2.1
Recruitment of phagocytes 1.2 2.2
Recruitment of myeloid cells 1.2 2.2
Failure of heart 2.0 2.2
Necrosis of muscle 1.6 1.9
Failure of heart 1.6 1.9
Necrosis of muscle -0.7 1.7
Accumulation of leukocytes 0.0 0.9
Migration of phagocytes 0.9
Binding of mononuclear leukocytes 1.2
Antimicrobial response 1.2
Flux of Ca2+ 1.1
Accumulation of myeloid cells 1.1
Accumulation of phagocytes 1.1
Ion homeostasis of cells 1.4
Adhesion of blood cells 1.4
Interaction of blood cells 1.4
Activation of blood cells 1.5
Systemic autoimmune syndrome 1.7
Cytotoxicity of natural killer cells 1.7
Apoptosis of muscle cells 1.6
Recruitment of leukocytes 1.7
Interaction of leukocytes 1.7
Cell death of immune cells 2.6
Cell death of lymphocytes 2.5
Cell death of fibroblasts 2.4
Apoptosis of leukocytes -0.4 2.2
z-score
3
-2.5

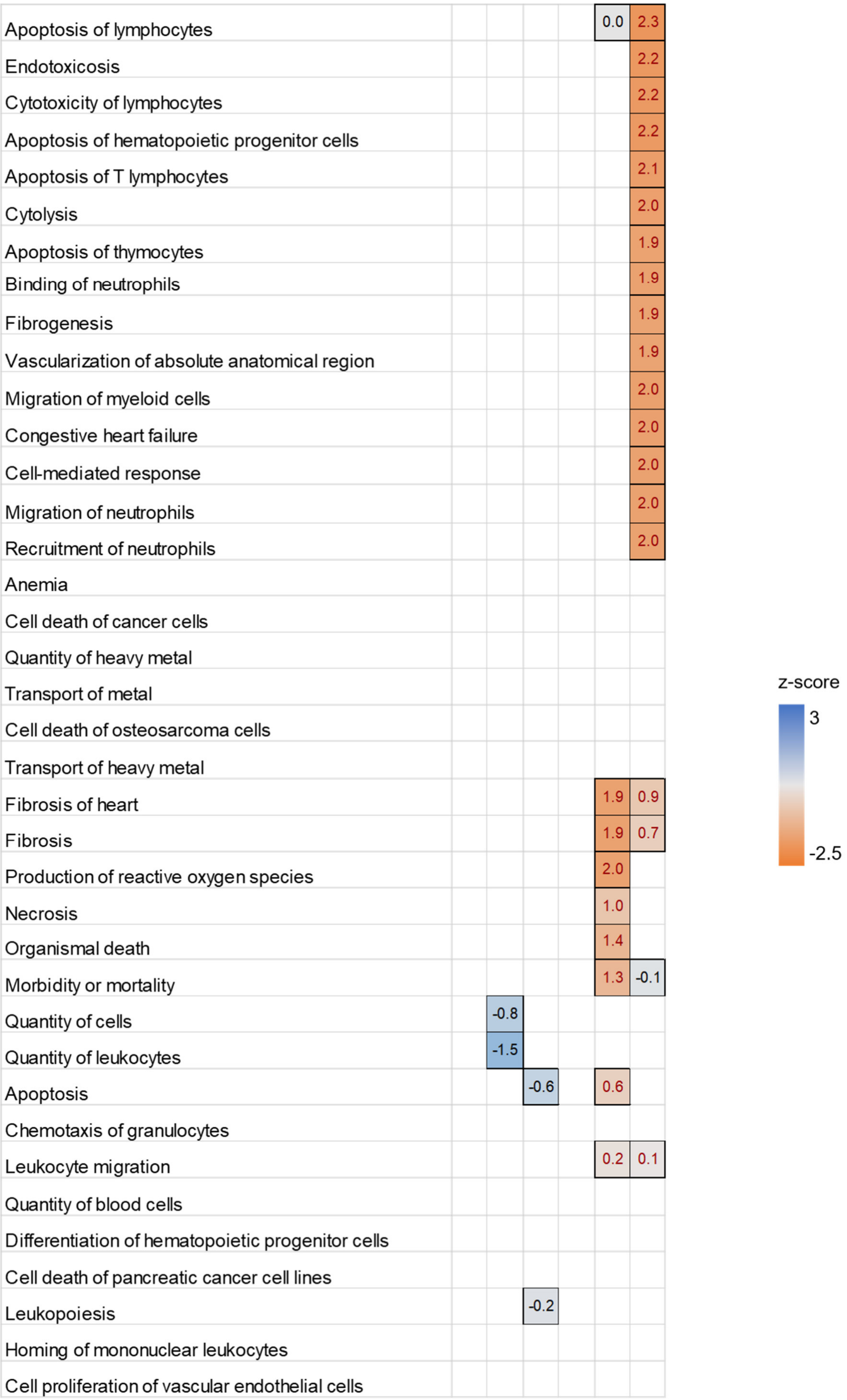

Apoptosis of lymphocytes
Endotoxicosis
Cytotoxicity of lymphocytes
Apoptosis of hematopoietic progenitor cells
Apoptosis of T lymphocytes
Cytolysis
Apoptosis of thymocytes
Binding of neutrophils
Fibrogenesis
Vascularization of absolute anatomical region
Migration of myeloid cells
Congestive heart failure
Cell-mediated response
Migration of neutrophils
Recruitment of neutrophils
Anemia
Cell death of cancer cells
Quantity of heavy metal
Transport of metal
Cell death of osteosarcoma cells
Transport of heavy metal
Fibrosis of heart
Fibrosis
Production of reactive oxygen species
Necrosis
Organismal death
Morbidity or mortality
Quantity of cells
Quantity of leukocytes
Apoptosis
Chemotaxis of granulocytes
Leukocyte migration
Quantity of blood cells
Differentiation of hematopoietic progenitor cells
Cell death of pancreatic cancer cell lines
Leukopoiesis
Homing of mononuclear leukocytes
Cell proliferation of vascular endothelial cells
0.0
2.3
2.2
2.2
2.2
2.1
2.0
1.9
1.9
1.9
1.9
2.0
2.0
2.0
2.0
2.0
1.9
0.9
1.9
0.7
2.0
1.0
1.4
1.3
-0.1
-0.8
-1.5
-0.6
0.6
0.2
0.1
-0.2
z-score
3
-2.5

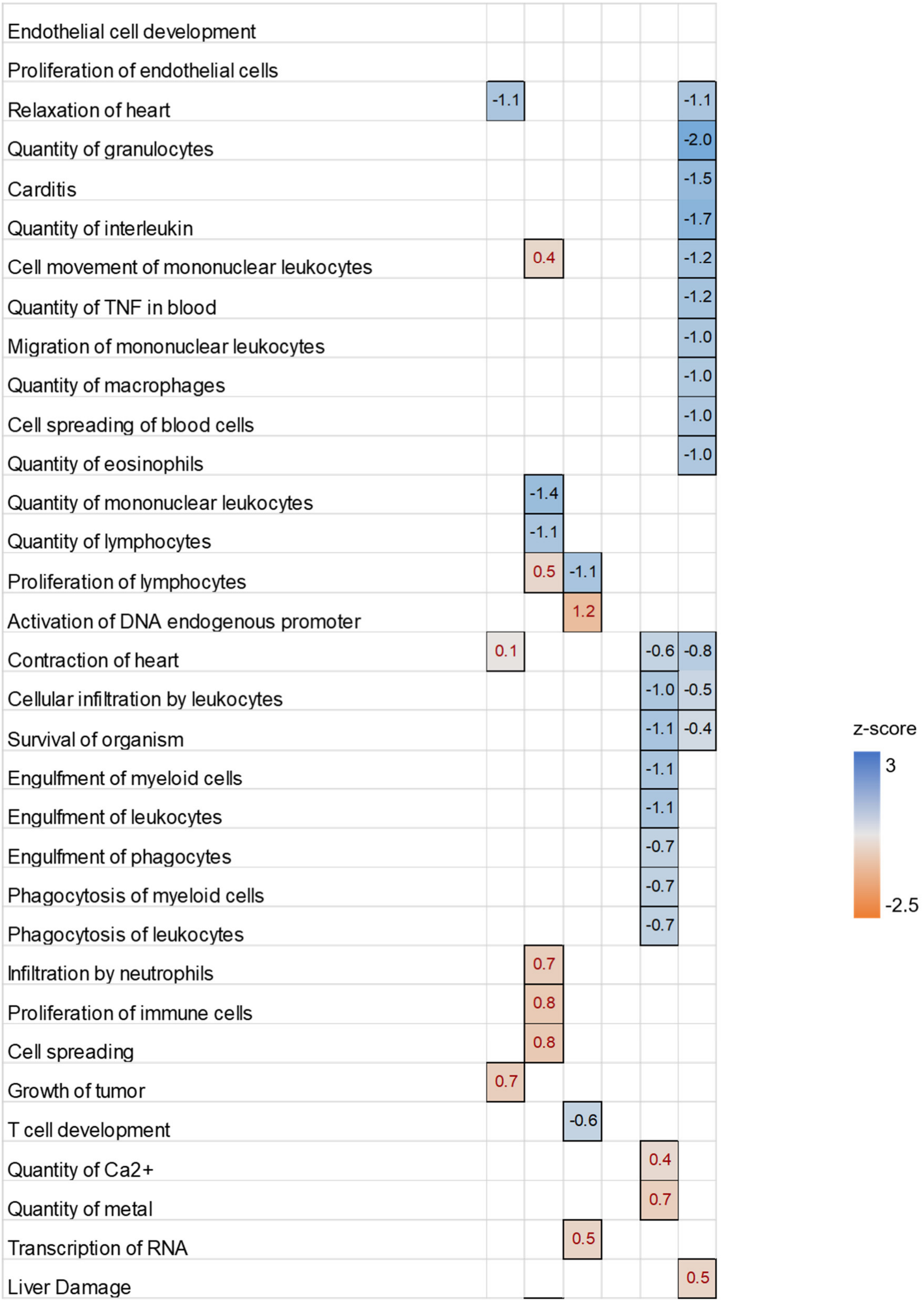

Endothelial cell development
Proliferation of endothelial cells
Relaxation of heart -1.1 -1.1
Quantity of granulocytes -2.0
Carditis -1.5
Quantity of interleukin -1.7
Cell movement of mononuclear leukocytes 0.4 -1.2
Quantity of TNF in blood -1.2
Migration of mononuclear leukocytes -1.0
Quantity of macrophages -1.0
Cell spreading of blood cells -1.0
Quantity of eosinophils -1.0
Quantity of mononuclear leukocytes -1.4
Quantity of lymphocytes -1.1
Proliferation of lymphocytes 0.5 -1.1
Activation of DNA endogenous promoter 1.2
Contraction of heart 0.1 -0.6 -0.8
Cellular infiltration by leukocytes -1.0 -0.5
Survival of organism -1.1 -0.4
Engulfment of myeloid cells -1.1
Engulfment of leukocytes -1.1
Engulfment of phagocytes -0.7
Phagocytosis of myeloid cells -0.7
Phagocytosis of leukocytes -0.7
Infiltration by neutrophils 0.7
Proliferation of immune cells 0.8
Cell spreading 0.8
Growth of tumor 0.7
T cell development -0.6
Quantity of Ca2+ 0.4
Quantity of metal 0.7
Transcription of RNA 0.5
Liver Damage 0.5
z-score
3
-2.5

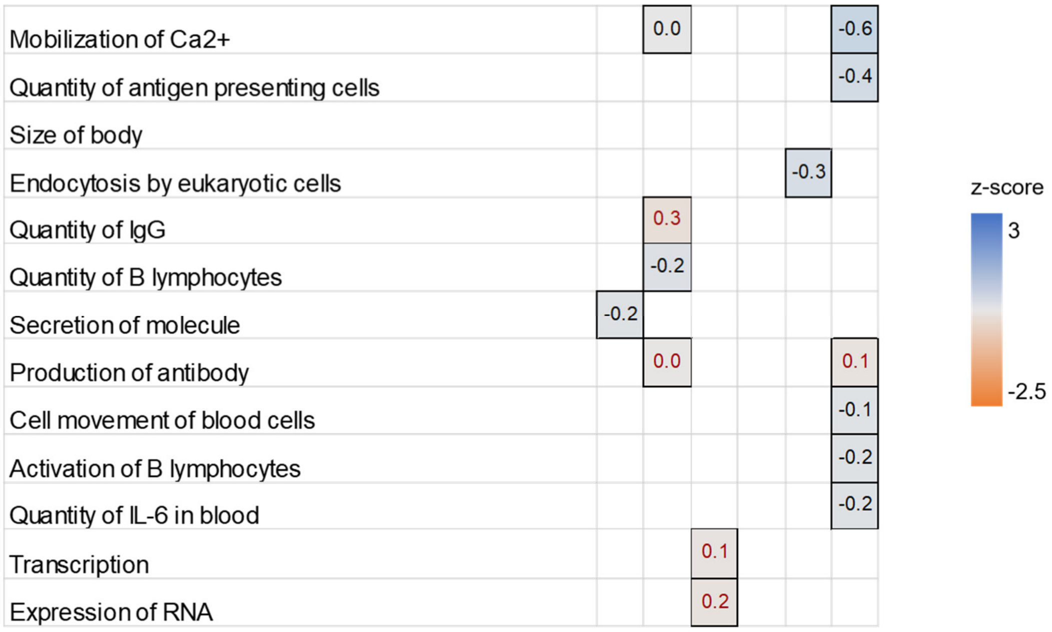
Mobilization of Ca2+
Quantity of antigen presenting cells
Size of body
Endocytosis by eukaryotic cells
Quantity of IgG
Quantity of B lymphocytes
Secretion of molecule
Production of antibody
Cell movement of blood cells
Activation of B lymphocytes
Quantity of IL-6 in blood
Transcription
Expression of RNA
0.0
-0.6
-0.4
-0.3
0.3
-0.2
-0.2
0.0
0.1
-0.1
-0.2
-0.2
0.1
0.2
z-score
3
-2.5

Material and Methods:

Animal study

Briefly, 128 adult male Fischer rats (strain-344/DuCrl) were purchased from Charles River Deutschland (Sulzfeld, Germany) and divided randomly into eight groups (control: n = 8, others: n = 5). They were treated daily by oral gavage with the PAs echimidine, heliotrine, lasiocarpine, senecionine, senkirkine, platyphylline or vehicle (0.15 M NaCl). The rats were maintained under conventional laboratory conditions.

For this study, gene expression analysis in lungs and kidneys of five randomly chosen animals of the control group and four animals of each treatment group dosed with 3.3 mg PA/kg body weight was investigated. After 28 days the animals were killed, organ weights were determined and tissue samples for transcriptomics analysis and histopathological examination were prepared. Shock-frozen samples of lungs, livers and kidneys were used for transcriptomics analysis. For histopathological pulmonary examination the left lung lobe was fixed in formaline (10 %), sliced in pieces with maximum 7 mm diameter and stained with hematoxylin and eosin.

Additionally, mice were treated with senecionine at the Leibniz Research Centre for Working Environment and Human Factors. Mice were treated once with 60 mg per kg body weight and killed after 12 h, 24 h, 36 h, 48 h and 72 h or with doses of 15, 30 and 60 mg per kg bodyweight and killed after 36 h. Lungs were extracted after the different time points.

Gene expression analysis

Gene expression analysis with RT-qPCR

The results of the microarray were verified by analyzing five randomly chosen genes (*Hrc, Rnase6, Tlr8, Stc1, Steap4, Hmgn5*) using RT-qPCR. These genes were identified to be significantly regulated in the whole genome microarray analysis of the lung samples.

2.4.1 cDNA synthesis and real-time PCR

In total, 1000 ng RNA from rat lungs was reverse transcribed into cDNA using the High capacity cDNA Reverse Transcription Kit (Applied Biosystems, Foster City, California, USA) with random primers and MultiScribe^{TM} Reverse Transcriptase. Thermal cycling conditions included 10 min at 25 °C, 120 min at 37 °C and 5 min at 85 °C. The resulting cDNA was diluted with 40 µl nuclease- free water.

A volume of 1 µl cDNA and 300 nM of each primer was used in a total volume of 10 µl with Maxima SYBR Green/ ROX qPCR Master Mix (Biozym, Oldendorf, Germany) for amplification in the Agilent technologies Aria DX Real-Time PCR System (Agilent, Santa Clara, California, USA). Primer sequences are listed in Table 1. The primers were designed with Primer 3 (https://bioinfo.ut.ee/primer3-0.4.0/) and quality was checked with the help of netprimer (http://www.premierbiosoft.com/netprimer/). The specific sequences for primer design were gained from the NCBI gene bank (https://www.ncbi.nlm.nih.gov/gene/).

Thermal cycling was initiated by a denaturation step at 95 °C, followed by 40 denaturation-primer annealing cycles for 0.5 min at 95 °C and 1 min at 60 °C, respectively. As last step, the template was elongated for 15 min at 60 °C before dissociation curves were recorded for verification of the effective amplification. The relative expression was calculated with the $2^{\Delta\Delta Ct}$-method (Livak and Schmittgen 2001). Thereby the C_t values were normalized to the mean of the housekeeping genes *Gapdh* and *Actb*.

Statistics were performed with SigmaPlot 14 by using one way ANOVA followed by Dunnett´s test and the definitions *p<0.05, ** p<0.01, ***p<0.001.

Table S4: Primer sequences for the verification of gene expression analysis data with RT-qPCR.

gene	forward primer (5`→ 3`)	reverse primer (5`→ 3`)
Actb	TGTGTTGTCCCTGTATGCCT	AGCGCGTAACCCTCATAGAT
Actc	CACGGCATTATCACCAACTG	AACAATGCCTGTGGTTCTCC
C3ar1	ATCTGCTCGTTGTGGATGATTAC	AGGAGGGTTGATTTCTGGTAGAT
Gapdh	GGCCGAGGGCCCACTA	TGTTGAAGTCACAGGAGACAACCT
Hmgn5	TACGGAGAGCTGCAACAATG	CACGACATTTGTGGTCTTCG
Hrc	CTTTCACCATTATCCCAAACCCA	TAGTTCTCATACTCCTGGGCATC
Lilrb3	CCGGGAAGAATATGCTGAATTGA	CTCATTTTCTGACATGGCTGAGT
Rnase6	GGTTTGAAATTCAGCACATATGGG	GTATCGGGTATAGTTGTTGACACC
Steap4	GCTTGACCATTGGATGCTTT	GCCAGGCAGTAGAGGAACTG
Stc1	GCTCATTGCCTTCCATCAAT	TCCAGCTGCCAGGACTACTT
Tlr8	CTCACCTACCTGCTGGCTTC	TGTCACACGTGCAGTCAAAA

Table S5: Results of differential gene expression analysis in lung for all treatments versus control conditions (See Excel sheet).

Bibliography

Livak KJ, Schmittgen TD (2001) Analysis of relative gene expression data using real-time quantitative PCR and the 2(-Delta Delta C(T)) Method. Methods 25(4):402-8 doi:10.1006/meth.2001.1262
